# Pigmentation and Vitamin D Metabolism in Caucasians: Low Vitamin D Serum Levels in Fair Skin Types in the UK

**DOI:** 10.1371/journal.pone.0006477

**Published:** 2009-08-03

**Authors:** Daniel Glass, Marko Lens, Ramasamyiyer Swaminathan, Tim D. Spector, Veronique Bataille

**Affiliations:** 1 Department of Twin Research and Genetic Epidemiology, Kings College London, St Thomas' Hospital Campus, London, United Kingdom; 2 Department of Chemical Pathology, Guys and St Thomas' NHS Foundation Trust, London, United Kingdom; 3 Dermatology Department, West Herts NHS Trust, Hemel Hempstead General Hospital, Herts, United Kingdom; University of Toronto, Canada

## Abstract

**Background:**

Vitamin D may play a protective role in many diseases. Public health messages are advocating sun avoidance to reduce skin cancer risk but the potential deleterious effects of these recommendations for vitamin D metabolism have been poorly investigated.

**Methodology/Principal Findings:**

We investigated the association between 25-hydroxy-vitamin D (25(OH)D), skin type and ultraviolet exposure in 1414 Caucasian females in the UK. Mean age of the cohort was 47 years (18–79) and mean 25(OH)D levels were 77 nmol/L (6–289). 25(OH)D levels were strongly associated with season of sampling with higher levels in the spring and summer months (p<0.0001). Light skin types (skin type 1 and 2) have lower levels of 25(OH)D (mean 71 nmol/L) compared to darker skin types (skin type 3 and 4) (mean 82 nmol/L) after adjusting for multiple confounders (p<0.0001). The trend for increasing risk of low vitamin D with fairer skin types was highly significant despite adjustment for all confounders (p = 0.001).

**Conclusions/Significance:**

Contrary to previous studies across different ethnic backgrounds, this study within Caucasian UK females shows that fair skin types have lower levels of 25(OH)D compared to darker skin types with potential detrimental health effects. Public health campaigns advocating sun avoidance in fair skinned individuals may need to be revised in view of their risk of vitamin D deficiency.

## Introduction

Sun exposure has long been associated with an increased risk of melanoma and non melanoma skin cancers as well as photo-ageing[1]. However, recent studies have shown that higher levels of 25(OH)D, which are maintained by sun exposure, are associated with lower incidence of many common diseases; especially cancer. Vitamin D has pleiotropic effects on many cell types and whilst most research has in the past focused on the impact on bone health, it is now clear that vitamin D deficiency affects many other systems [2]. Vitamin D deficiency has been linked to cancer, inflammatory disorders, autoimmune and psychiatric diseases [3], [4], [5], [6]. More recent studies have also shown an association between low serum 25(OH)D levels and metabolic syndrome characterised by abdominal obesity, raised blood pressure and triglycerides with low HDL cholesterol [7].

Vitamin D serum levels are influenced mainly by sun exposure and to a lesser extent by diet[8]. Advice about avoiding sun exposure and the use of sunscreens for the primary prevention of skin cancer may lead to vitamin D deficiency in susceptible individuals. Hence public health campaigns for skin cancer prevention may need to be reviewed to be adapted to respective geographical areas in view of the variations in skin types and ambient ultraviolet exposure.

Vitamin D levels are heritable with twin studies showing 43% of 25(OH)D and 65% of 1,25-hydroxyvitamin D variability is genetically determined [9]. Skin type (or skin colour) influences vitamin D levels, larger amounts of melanin in darker skin compete for UVB photons with 7-dehydrocholesterol which is the substrate for making vitamin D[10]. However, studies of the relationship between skin type and Vitamin D level has mainly compared extreme ethnic backgrounds [11], [12], [13] rather than within Caucasian cohorts. The influence of sun exposure and sunbed exposure on vitamin D serum levels within Caucasian populations is also poorly studied. This study's aim was to investigate the relationship between vitamin D serum levels, sun exposure and skin type in a large population based study in the UK.

## Results

### Age, season and vitamin D

Mean age of the self-classified European Caucasian women was 47 years (range 18–79). Mean 25(OH)D levels were 77 nmol/L (median 71 nmol/L, range 6–289 ). Vitamin D levels were influenced by season of blood sampling (r = 0.34 p<0.0001): mean 25(OH)D level during the spring and summer months was 93 nmol/L compared to 66 nmol/L in the autumn and winter months. Mean BMI was 25 (range 17–51) and was weakly inversely associated with 25(OH)D (r −0.04, p = 0.045). However, the lowest levels of 25(OH)D were seen at the extremes of BMI as women with BMI below 19 had a mean level of 67 nmol/L and women with BMI above 30 had a mean level of 68 nmol/L. Vitamin D levels did decrease with age but this did not reach statistical significance.

### Skin type, sun exposure

The distribution of skin types is shown in Table 1 with the majority of the subjects belonging to skin type 2 and 3. Mean number of weeks of holiday abroad over a lifetime was 21 weeks (range 0–318) and was positively correlated with skin type with a highly significant trend (p<0.0001): darker skin types (3 and 4) had on average 23 weeks of holidays abroad compared to 18 in those with fairer skin types ( skin type 1 or 2) ( Table 1). Ever use of sunbed was reported in 44% of the subjects and inversely correlated with age (p<0.0001). Skin type was also correlated with sunbed: 52% of the twins with dark skin types used sunbeds compared to 39% in those with fairer skin types (p<0,0001).

**Table 1:** Odds ratios for vitamin D levels equal or below 70 nmol/L according to skin type (adjusting for age, BMI, season of sampling, total number of weeks of sunny holidays and sunbed use).

Skin type	Nos (%)	Odds ratios	P value	Confidence Intervals
Skintype 1	169(16)	1.0		referent
Skintype 2	432(33)	0.85	0.507	0.54–1.36
Skintype 3	469(36)	0.59	0.033	0.37–0.96
Skintype 4	253(19)	0.45	0.002	0.27–0.75

Test for trend. Chi Square (one degree of freedom): 10.35 p = 0.0013.

### Vitamin D and ultraviolet light exposure

25(OH)D levels were positively associated with sunbed exposure (p = 0.009) and total number of weeks on holiday abroad (p = 0.0344) after adjusting for age, skin type and season of sampling. Vitamin D serum levels were divided into two groups with a cut off chosen at 70 nmol/L which was the median 25(OH)D level and is approaching the minimal 25(OH)D level needed for maximal suppression of parathyroid hormone [14], [15] [16]. Sunbed use was reported in 52% of the high vitamin D group (above 70 nmol/L) compared to 40% of the low vitamin D group (equal or below 70 nmol/L) (p<0.0001). More than 25 weeks of holidays abroad in a sunny climate over a lifetime was reported in 37% of the high vitamin D group compared to 30% of the low vitamin D group (p = 0.014). This difference remained significant after adjusting for age, skin type and season of sampling (p = 0.034). Mean number of sunburns was 2.1 (range 0–67). Subjects with 2 or more sunburns over a lifetime had a mean vitamin D serum levels of 71 nmol/L compared to 79 nmol/L in those with less than 2 sunburns. This association between a history of sunburns and low levels of vitamin D remained significant despite adjusting for age (p = 0.04).

### 25-OH-Vitamin D and skin type

Skin type and 25(OH)D levels were significantly and positively associated (p<0.0001) and remained so after adjustment for all confounders. Table 1 shows the trend in risk for low 25(OH)D levels (defined as equal or below 70 nmol/L) in relation to skin type. The decreasing trend in risk from skin type 1 (light) to skin type 4 (dark) was highly significant with an odds ratio of 0.45 for subjects with skin type 4 (Chi Square: 10.35, p = 0.001 after adjustment for age, season of sampling, BMI, total number of weeks abroad and sunbed use).

**Figure 1:**
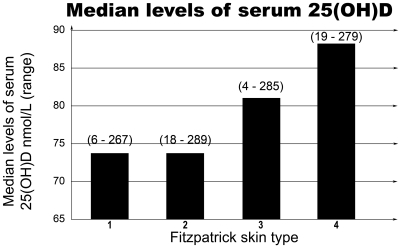
Median levels of serum 25(OH)D for each skin type.

Figure 1 shows the median levels of serum 25(OH)D according to skin type. 45% of skin type 1 subjects had low serum 25(OH)D compared to 28% in the skin type 4. When winter 25(OH)D levels were measured, the differences were more marked- low 25(OH)D levels were found in 60% of the skin type 1 compared to 38% of the skin type 4 (p<0,0001). Clinical vitamin D deficiency defined as equal or below 30 nm/L was found in 10% of skin type 1 compared to 5% of skin type 4. However the number of subjects with levels equal or below 30 nm/L was small and this did not reach statistical significance (p = 0.07). 25(OH)D serum levels were not affected by place of residence- urban versus rural or latitude (North UK versus South UK). Social class derived from post codes and divided in quintiles was not associated with 25(OH)D serum levels.

## Discussion

This population based study in Caucasian females shows 25(OH)D levels are suboptimal in a large percentage of fair skinned females in the UK. Although this study did not record sun exposure in the weeks preceding the blood sampling, the data on sun exposure and sunbed exposure does reflect a sun seeking behaviour which was shown to correlate with vitamin D levels. Adjusting for the season of sampling did in part address the fact that ambient UV radiation at the time of sampling may have varied significantly between subjects. Studies across different ethnic groups have previously shown that darker skin types especially African Americans and Asians have the lowest vitamin D levels but these studies often compared skin types across ethnic backgrounds in different continents [11], [12], [13], [17], [18]. Many of these studies have also investigated dark skinned individuals migrating to temperate climates. Genetic, diet and cultural variations may explain different vitamin D levels in Caucasian and non-Caucasian groups. African American women have much lower levels of serum vitamin D than white women but yet have much higher bone density and fewer fractures than white women so the relationship between bone health and vitamin D levels is complex and is highly dependent on ethnicity [19]. Melanin is thought to limit penetration of UVR and result in less production of vitamin D in darker skin in Africans and Asians.

Contrary to current beliefs based mainly on studies between different ethnic groups, this study shows that fair skin types within a Caucasian population in the UK are most at risk of vitamin D deficiency. This may be due to sun avoidance or may result from genetic differences in vitamin D metabolism in subjects with fair skin compared to those with darker skin. A study of more than 1000 subjects in France linked fair skin to low vitamin D. This was hypothesised to result from sun avoidance behaviour in fair skinned subjects [20]. However collection of sun exposure data over a lifetime can be subject to poor recall. In this latter study, collection of sun exposure data was based on a global assessment of self reported sun exposure divided in 4 categories from none to high without details on sun seeking activities such as number of weeks of holidays in hot climates and use of sunbed. The skin type data is not based on the the Fitzpatrick classification so cannot easily be compared to our study. The French data also contained photo-type 5 and 6 which means that this study was not confined to Caucasians and the numbers were not given for each photo-type so again comparisons are difficult.

Primary prevention campaigns for skin cancers have been very active for the last 15 to 20 years in the UK and advocate sun avoidance especially for fair skinned individuals. Sun avoidance may result in low vitamin D levels especially during winter. Australian UV levels are higher than those in the UK, but vitamin D deficiency is still prevalent there [21]. Bone health and skin colour have been also been linked in studies showing that UK women with fair skin have an increased risk of osteoporosis compared to those with darker skin.[22]. Furthermore, Bruyere et al [23] showed in more than 8000 osteoporotic women across Europe, those in Northern Europe had lower levels of vitamin D compared with women in Southern Europe. This reduced risk of osteoporosis in Southern Europe may be due to higher levels of UVR or diet, but may also be explained by darker skin types.

Dietary vitamin D comes mainly from oily fish and there is no evidence that skin type, within a Caucasian population, would influence diet significantly especially in the UK and act as a significant confounder. Examining the association between vitamin D supplementation, vitamin D levels and skin type was not possible in the study presented here as vitamin D supplementation data was incomplete at the time of blood sampling and only available on a small subset of twins. However, the prevalence of regular vitamin D supplementation in 4265 subjects within the Twins UK cohort was generally low (4.69%).

In concurrence with other groups, sunbed exposure and holidays abroad were associated with increased levels of 25(OH)D levels in the study reported here[24]. This confirms the importance of ultraviolet light in maintaining adequate levels of vitamin D in temperate climates such as the UK. The negative association between number of sunburns and 25(OH)D level also confirms that sun sensitive subjects (mainly skin type 1 and 2) are most likely to have vitamin D deficiency. Suboptimal levels of vitamin D were found in more than 60% of those with skin type 1 in winter. Furthermore 10% of subjects with skin type 1 had clinical vitamin D deficiency (≤30 nmol/L) which would require treatment. Adjusting for sun exposure and sunbed exposure did not completely remove the association between skin type and vitamin D, so factors other than sun may be implicated.

Further research is required to explore potential genetic links between low vitamin D levels and pigmentation. Polymorphisms in the vitamin D binding protein and vitamin D hydroxylase ( CYP2R1) genes influence vitamin D levels although vitamin D receptor (VDR) genotypes have not been consistently linked to vitamin D levels in healthy individuals [25]. In prostate cancer patients, the Fok1 ff VDR polymorphism when associated with low plasma vitamin D levels conferred the highest risk of prostate cancer in the USA as well as giving rise to more aggressive tumours [26]. The anti-proliferative and differentiation effects of vitamin D on many cell types have long been known with therapeutic targets such as psoriasis and cancer but the effects of vitamin D are very complex with the involvement of many pathways in the cell cycle [27]. It is becoming apparent that skin pigmentation via the MSH pathway is involved in cell survival and proliferation as well as inflammation so it is possible that pigmentation genes and genes involved in vitamin D metabolism may be linked [28].

Considering the low levels of vitamin D in Caucasian females in the UK, the current sun avoidance advice in fair skinned individuals in Northern Europe resulting in lower vitamin D levels may have deleterious effects on other diseases related to cancer and inflammation and need to be studied in other Caucasian populations. Furthermore, optimal levels of serum vitamin D have, to date, mainly been determined in terms of parathyroid hormone (PTH) and calcium regulation as well as bone health, but less data is available in relation to the prevention of other diseases such as cancer. It has been roughly estimated that exposure of the face and arms for 5 to 15 minutes (this being very variable depending on skin type and latitude) between 10 am and 3 pm in the spring, summer and autumn may be sufficient to provide a daily dose of 1000 IU of cholecalciferol in the UK. However, as most public health education on the prevention of skin cancer recommends avoiding sun exposure between 11 am and 3pm and use of sunscreens at all time, it is likely strict sun avoidance may further contribute to vitamin D deficiency [29], [30]. Vitamin D supplementation could provide a long term solution, but in this study which examines a random cross section of UK population, the percentage of subjects currently taking vitamin D supplementation is very low. In addition there is no added vitamin D in fresh milk or other food in the UK which differs from the USA and Canada, sun remains currently a fast, free and easy source of vitamin D in Europe.

Skin pigmentation is a very variable trait which has evolved slowly presumably providing survival advantages during migrations [31]. Fair skin types have evolved very slowly to maximise vitamin D production following the limited sun exposure with migration to temperate climates so reducing sun exposure drastically may have detrimental effects as it may affect this adaptive evolution of human skin pigmentation. Further research in this field will not only help with primary prevention messages in relation to skin cancer prevention but is also likely to provide further insights on the possible interactions between pigmentation genes, genes involved in cell cycle regulation and vitamin D metabolism.

## Material and Methods

Ethics Statement: Ethics Committee approval for this study was obtained at the Guy's and St Thomas Hospital Trust, London. Subjects were not aware of the hypotheses being tested as they were part of a large study investigating many age related diseases and traits for which informed written consent was obtained. (www.twinsuk.ac.uk).

2786 consecutive women (752 monozygotic twins (MZ) and 2034 dizygotic twins (DZ)) were recruited from the Twins UK Adult Twin Registry in London (wwww.twinsuk.ac.uk) between January 1997 and December 2000. The twins involved in this study have previously been shown to be representative of the UK singleton population in general [32] and for this study were treated as population based individuals. All were adult females aged between 18 and 79 years of age (mean age 47). For historical reasons, the register consists mainly of females. Subjects were not aware of the hypotheses being tested as they were part of a large study investigating many age related diseases and traits (wwww.twinsuk.ac.uk). As well as answering a comprehensive questionnaire on many diseases other than skin diseases, the women were given a validated questionnaire on sun and sunbed exposure. Sun exposure was assessed with number of weeks of holiday abroad over a lifetime. Sunbed exposure was defined as ever using sunbeds over a lifetime. Sunburn episodes were recorded by 10 years age group over a lifetime. Total number of sunburns was created by adding the number of sunburns over a lifetime. Sunburn was defined as a burn severe enough to cause redness, for more than 2 days, peeling or blistering. The data was collected with the aim of gauging the amount of sun, subjects were exposed to throughout their life i.e. Their lifetime UVR habits rather than a snap shot of sun exposure prior to the 25(OH)D blood test. Social class was derived using postcodes which allowed classification of the twins using an Index of Multiple Deprivation for England (IMD) –give ref or more details. Residence in rural versus urban areas was also collected. Latitude of place of residence within the UK was also recorded.

A skin examination was performed by trained research nurses and included a record of skin type using the Fitzpatrick classification. Fair skin grouped Fitzpatrick skin type 1 and 2 whilst dark skin included skin type 3 and 4.

Total 25-hydroxy vitamin D levels were measured by radioimmunoassay using Diasorin RIA kit (Diasorin, Minnesota, USA). This assay has a detection limit of 4 nmol/L and the analytical coefficient of variation (CV) of the method is 9.1% at 22 nmol/L. Of the 2786 female twins, 1414 had both skin data and data on serum levels of vitamin D so the analyses in this manuscript are restricted to these 1414 twins. Low 25(OH)D levels were defined as equal or below 70 nmol/L which is the median level in this population and approaching the minimal 25(OH)D level needed for maximal suppression of parathyroid hormone [15]. Clinically relevant low vitamin D levels were defined as equal or below 30 nmol/L as this is critical level for parathyroid hormones increase [33]. Serum vitamin D binding protein was also measured by an immuno-pelometric assay. The detection limit was 50 mg/l and the intra and interassay CV at 250 mg/l was 2% and 3.8% respectively but was only available in a subset of 385 subjects [9].

### Statistical methods

All statistical analyses were performed using the Stata statistical software (Stata Corp, USA). Associations between 25(OH)D levels and skin type, age, season of sampling, BMI, sun exposure, number of weeks abroad and sunbed use were investigated using Pearson correlations and regressions. For categorical data, chi square was used. Standard linear regressions were used to look at the association between serum 25(OH)D and other variables. Logistic regression was used for calculating risk of low 25(OH)D serum levels in relation to skin type and sun exposure with adjustment for age and season of sampling. All analyses were adjusted for the fact that the twin pairs were related either using the command cluster in logistic regressions or the command xtgee in Stata.
